# Unmasking Heterogeneity: Evaluating Distinct Sepsis Clinical Phenotypes and Their Association With Mortality in Critical Adult Patients

**DOI:** 10.7759/cureus.102244

**Published:** 2026-01-25

**Authors:** Júlia A Silva, Fabio F Neves

**Affiliations:** 1 Medicine, Federal University of São Carlos, São Carlos, BRA

**Keywords:** clinical phenotype, critical care, mortality, sepsis, septic shock

## Abstract

Objective: This study aimed to evaluate three phenotypic classifications based on their ability to predict mortality in critically ill adult patients with sepsis.

Methods: This single-center cohort study involved 106 patients diagnosed with sepsis upon admission to the intensive care unit (ICU). The patient population was categorized according to three distinct clinical phenotypic models: the first based on age and thermal profiles, the second stratifying patients into four specific groups (multiple organ failure, respiratory dysfunction, neurological dysfunction, and miscellaneous), and the third assessing arterial blood pressure trajectories within the initial 10 hours of ICU stay. Multiple logistic regression models were fitted to evaluate the utility of these classifications in predicting in-hospital mortality. The performance of a model incorporating the three phenotypic classifications was compared with the acute physiology and chronic health evaluation II (APACHE II) and sequential organ failure assessment (SOFA) scores.

Results: It was observed that elderly patients presenting with hypothermia showed a significantly higher mortality risk compared to younger, normothermic, or febrile subjects (OR 17.32 [95% CI 1.95-153.14], *p* = 0.010). Furthermore, patients with multi-organ failure presented a significantly higher risk of death (OR 5.87 [95% CI 1.17-29.94], *p* = 0.031). Finally, persistent hypotensive individuals were not found to have a significantly elevated risk of death (*p* = 0.300). The final predictive model’s area under the ROC curve was 0.856, which was not inferior to that of APACHE II (0.776) or SOFA (0.764) in the sample studied.

Conclusions: When used together, the phenotypically analyzed classifications demonstrated good accuracy in predicting mortality among critically ill patients with sepsis.

## Introduction

Sepsis is defined as a life-threatening organ dysfunction resulting from a dysregulated host response to infection [1]. In particularly severe cases, this condition can progress to septic shock, which is associated with a significantly high mortality risk. The considerable global impact of this syndrome was highlighted by the Global Burden of Disease Study, which estimated a worldwide sepsis incidence of 48.9 million cases and 11.0 million sepsis-related deaths in 2017 [2].

The pronounced phenotypic heterogeneity of sepsis patients presents significant therapeutic challenges, moving beyond the traditional "one-size-fits-all" approach. This variability complicates the accurate diagnosis, prognosis, and optimal management of the condition [3].

Comorbidities significantly influenced the trajectory and outcomes of sepsis. Chronic conditions, such as severe liver dysfunction, chronic kidney failure, and cardiovascular diseases, compromise the host's ability to develop an effective response to infection [4,5]. Conversely, studies have indicated that mild-to-moderate obesity often confers a more favorable prognosis than normal weight or malnutrition [6]. This paradox is attributed to the protective role of the adipose tissue, which serves as a crucial metabolic reserve during the acute phase response and is hypothesized to modulate immune function and support thermogenesis against severe infections [7].

In addition to comorbidities, age was another critical source of heterogeneity. Elderly individuals commonly exhibit an attenuated febrile response due to diminished production of pyrogenic cytokines (e.g., IL-6, TNF-α), reduced muscle mass, and frequent neurological comorbidities [8]. Notably, hypothermia was not correlated with elevated mortality in the older population. In contrast, younger patients with sepsis who developed hypothermia had significantly higher mortality rates. These age-dependent differences in sepsis outcomes related to thermoregulatory failure may be partially explained by the propensity of younger individuals to develop exacerbated immune responses, specifically hyperinflammation, which can lead to immune exhaustion. Supporting this, a multi-cohort study by Baek et al. found that hypothermic non-elderly sepsis patients had a 90-day mortality rate of 27.4%, which was substantially higher than the rates of 19.6% and 11.9% reported for the normothermia and hyperthermia groups, respectively [9].

In addition to age and underlying health, variability in sepsis is further driven by clinical presentation, specifically in the affected organs. Machine learning models identified four distinct clusters of organ dysfunction in 2533 patients with sepsis: shock with elevated creatinine, minimal multi-organ dysfunction syndrome, shock with hypoxemia, altered mental status, and hepatic disease [10]. Mortality rates varied significantly across these clusters (11%, 12%, 28%, and 21%, respectively), revealing prognostic differences beyond traditional predictors, such as the sequential organ failure assessment (SOFA) score and acute physiology and chronic health evaluation (APACHE II) [10]. Similarly, a single-center cohort study by Shald et al. analyzed 320 patients with sepsis and categorized them into four clinical phenotypes: multi-organ failure (MOF), respiratory dysfunction (RD), neurological dysfunction (ND), and other patients (OP) [11]. The in-hospital mortality rates varied widely as follows: MOF (48.4%), ND (39.7%), RD (20.8%), and OP (13.7%).

Hemodynamics is another crucial clinical factor for phenotyping critically ill patients with sepsis. Specifically, a cluster-based approach that focuses on the initial response to treatment may be vital for improving patient outcomes. Zhu et al. investigated patient clustering based on systolic blood pressure (SBP) variability and identified seven distinct SBP trajectories within the first 10 h of Intensive Care Unit (ICU) admission [12]. Their findings revealed that patients exhibiting an increasing SBP trend from 140 mmHg had the lowest mortality rate (11.8%). Conversely, a persistently low SBP was associated with the worst prognosis, with a mortality rate of 40.5%. This highlights the clinical importance of monitoring blood pressure trends to accurately identify high-risk patients.

Finally, variability in sepsis is also attributable to the site of infection and specific bacterial etiology. Epidemiological studies have identified lower respiratory tract infections as the most prevalent source of infection, correlating with the highest mortality rates [13,14]. Poor prognosis is also frequently observed in cases involving gram-negative bacteria, especially those exhibiting carbapenem resistance [15].

The phenotype-based approach to sepsis management offers significant promise, opening new avenues for clinical research and paving the way for the development of precision medicine. Although observational studies have successfully demonstrated the utility of this approach, they rely primarily on biomarkers with limited availability and complex AI-based algorithms. The complexity of these models presents a substantial obstacle for clinicians to effectively apply them at the bedside. This study aimed to assess three distinct and readily applicable clinical phenotypic classifications based on their prognostic utility for mortality in patients with sepsis.

## Materials and methods

Study design

This retrospective cohort study enrolled adult patients admitted to the intensive care unit (ICU) with sepsis at the University Hospital of São Carlos, Brazil, between January 2021 and December 2023. Sepsis was diagnosed in accordance with the Third International Consensus Definition for Sepsis and Septic Shock (Sepsis-3) [1].

Individual patient data were retrieved from the university hospital management applications. Variables were primarily extracted from the initial vital signs, clinical neurological examinations, and laboratory values recorded upon admission to the ICU. The comprehensive dataset included demographics (age, sex, ethnicity, and weight); markers of disease severity and comorbidities, such as SOFA score, APACHE II score, and comorbidities used to calculate the Charlson Comorbidity Index (CCI); and specific clinical information (primary diagnosis, Glasgow Coma Score (GCS), laboratory results, use of mechanical ventilation, need for hemodialysis, and vasopressor use). The SOFA and APACHE II scores were calculated using the worst values recorded within the first 24 hours after admission to the ICU.

Following the compilation of these data, patients were categorized into three distinct clinical phenotype classifications, and the following study outcomes were assessed: in-hospital mortality, total length of hospital stay, and duration of ICU stay.

To evaluate the prognostic utility of the three phenotypic classifications, the study population was stratified into two distinct groups based on the primary outcome: survivors and non-survivors. This comparative approach allowed for a detailed assessment of how baseline clinical characteristics, disease severity markers, and the specific phenotypic models are associated with in-hospital mortality.

The study protocol adhered to the principles of the Declaration of Helsinki and was approved by the Federal University of São Carlos Ethical Board (Approval No. 7838.5424.6.0000.5504).

Phenotypic classifications

Three distinct clinical phenotypic classifications were selected for evaluation, involving the analysis of specific high-risk profiles: advanced age with hypothermia, presence of organ failure, and SBP curve within the first 10 h of ICU admission.

The selection of these phenotypic classifications was strategically guided by the need for prognostic models that are readily applicable at the bedside. While numerous sepsis phenotypes have been identified through complex biomarkers and advanced artificial intelligence algorithms, their practical utility is often hindered by limited availability in standard clinical settings.

These classifications were used to stratify the patient cohort. Details and definitions of the three clinical phenotypic classifications are summarized in Table 1. 

**Table 1:** Clinical phenotypic classifications utilized in the study SBP: Systolic blood pressure; MOF: Multi-organ failure; RD: Respiratory dysfunction; ND: Neurologic dysfunction; OP: Other patients.

Study	Phenotypic Classification
Baek et al. [9]	Cluster A - older age and lower body temperature
	Cluster B - younger age and wide range of body temperature
	Cluster C - higher body temperature than Cluster A
Shald et al. [11]	MOF - multi-organ failure
	RD - respiratory dysfunction
	ND - neurologic dysfunction
	OP – other patients
Zhu et al. [12]	Class 1 - SBP steady about 100 mmHg
	Class 2 - Stable SBP change trend, mean value 82 mmHg
	Class 3 - SBP gradually increased from 140 mmHg
	Class 4 - SBP steadily increasing from 110–120 mmHg to 120–130 mmHg
	Class 5 - SBP rapidly decreasing from 130 mmHg to 100 mmHg
	Class 6 - SBP rapidly decreasing from 150–160 mmHg to 110–120 mmHg
	Class 7 - SBP initially on an increasing trend, followed by decreasing. Average SBP > 160 mmHg

To align our current cohort with the sepsis clusters previously defined using artificial intelligence by Baek et al. [9], we established two specific parameters: lower body temperature was defined as a temperature less than 36°C, and older age was defined as an age exceeding 65 years.

The second classification, adapted from Shald et al. [11], was defined using the following criteria: the multi-organ failure (MOF) phenotype included patients presenting with a SOFA score > 6 and a GCS score > 9. The respiratory dysfunction (RD) phenotype comprised patients with a SOFA score ≤ 6, a GCS score > 9, and a requirement for invasive or noninvasive positive pressure ventilation. Conversely, the neurologic dysfunction (ND) phenotype was defined by a GCS score ≤ 9. Patients who did not meet the criteria for any of the three aforementioned phenotypes were categorized as having the Other Patients (OP) phenotype.

Finally, the data necessary for the Zhu et al. [12] classification were extracted from automatically measured SBP values recorded over 10 h following ICU patient admission.

Statistical analyses

Differences between groups for categorical data were analyzed using the chi-square test, with results reported as frequencies and percentages. Conversely, continuous nonparametric data were compared using the Mann-Whitney U test and presented as medians with corresponding minimum and maximum ranges. For comparisons involving more than two groups, the Kruskal-Wallis test was employed.

Multiple logistic regression was used to assess the association between the three distinct phenotype classifications and risk of in-hospital mortality. All models were adjusted for essential covariates, selected based on the literature: age, sex, ethnicity, weight, site of infection, and Charlson Comorbidity Index (CCI). Additionally, a stepwise approach with forward selection included variables that achieved a statistical significance level of p < 0.10 in the univariate analysis. This strategy led to the incorporation of additional variables into the model, including the need for hemodialysis and serum lactate, PaCO2, and hemoglobin levels.

A final predictive model incorporating the three phenotypic classifications was developed. The predictive accuracy of this model for in-hospital mortality was assessed using a receiver operating characteristic (ROC) curve analysis. Specifically, the area under the curve (AUC) and overall model accuracy were calculated and directly compared with the established performance metrics of SOFA and APACHE II scores.

Statistical significance was defined as a probability value of p < 0.05. All statistical analyses were conducted using Jamovi statistical software (version 2.6), which utilizes the R language engine.

## Results

Baseline characteristics of the participants

A total of 106 patients with sepsis were included in this study, with a median age of 65.5 years (range, 19-93 years) and a median weight of 66.2 kg (range, 40-165 kg). The median Charlson Comorbidity Index (CCI) in this cohort was 4 (range, 0-11). Demographically, the group was predominantly male, comprising 62 participants (58.5%); 68 patients (64.1%) self-identified as white. The most common site of infection was the lungs, followed by the urinary tract, affecting 58.5% and 22.6% of the cases, respectively. The study group exhibited high disease severity, as reflected by a median SOFA score of 7 (range, 2-17) and a median APACHE II score of 16.5 (3-44). A large majority of the patients developed septic shock, defined as the continuous requirement for vasoactive drug support, which affected 85.8% of the cohort. Furthermore, mechanical ventilation and hemodialysis were necessary interventions for 58.5% and 20.0% of patients, respectively. Overall, the in-hospital mortality rate in this population was 34.9%.

When comparing survivors and non-survivors (Table 2), we observed no significant differences in baseline characteristics, including age, sex, ethnicity, body weight, comorbidities, initial severity scores, and laboratory test results. However, nonsurvivors required mechanical ventilation and renal replacement therapy (hemodialysis) more frequently than survivors. Furthermore, non-survivors had a substantially higher prevalence of pneumonia (83.7%) than survivors (44.2%).

**Table 2:** Baseline characteristics of 106 septic patients admitted to ICU, stratified by mortality status ICU: Intensive care unit; SOFA: Sequential organ failure assessment; APACHE II: Acute physiology and chronic health evaluation II. Inter-group differences for continuous data were determined by the Mann-Whitney test and reported as U. For categorical data, the chi-square test was employed, with results expressed as X2 followed by the degrees of freedom in brackets.

Variables	Survivor (n = 69)	Non-survivor (n = 37)	Statistics	p-value
Demographics				
Age (years)	65 (19 – 90)	69 (32 – 93)	1055	0.142
Male – n (%)	38 (55.0)	24 (64.8)	0.951 [1]	0.329
White – n (%)	43 (62.3)	25 (67.5)	0.289 [1]	0.591
Weight (kg)	68.4 (43.2 – 145.0)	66.1 (40.0 – 165.0)	1209	0.824
Charlson Comorbidity Index	4 (0 – 11)	4 (0 – 11)	1185	0.544
Prognostic markers				
SOFA	6 (2 – 17)	7 (2 – 15)	1098	0.234
APACHE II	16 (3 – 44)	18 (3 – 30)	1014	0.082
Lactate (mmol/L)	2.40 (1.00 – 6.63)	2.55 (1.00 – 13.70)	1090	0.147
Hemoglobin (g/dL)	11.8 (3.7 – 15.4)	12.8 (5.9 – 17.7)	979	0.057
Platelets (10^3^/µL)	214 (13 – 690)	203 (14 – 466)	1251	0.854
Mechanical ventilation – n (%)	17 (24.6)	19 (51.3)	7.66 [1]	0.006
Hemodialysis – n (%)	7 (10.1)	13 (35.1)	10.9 [2]	0.004
Vasoactive drugs use – n (%)	48 (69.5)	30 (81.0)	1.64 [1]	0.200
Infection sites				
Lung – n (%)	31 (44.2)	31 (83.7)	16.8 [5]	0.005
Urinary – n (%)	21 (30.4)	3 (8.1)		
Skin and subcutaneous – n (%)	9 (13.0)	2 (5.4)		
Others – n (%)	8 (11.5)	1 (2.7)		
Outcomes				
ICU length of stay (days)	7 (1 – 147)	9 (1 – 54)	1158	0.431
Hospital length of stay (days)	15 (2 – 147)	13 (1 – 66)	1082	0.197

Finally, no significant differences were observed between the groups in the secondary outcomes of ICU length of stay and length of hospital stay.

Performance of the phenotypic classification in the prediction of in-hospital mortality in septic patients

Significant differences were observed between survivors and non-survivors in the first two phenotypic classifications (Table 3). However, no statistically significant differences were observed in blood pressure curves.

**Table 3:** Frequency of clinical phenotypic classifications, stratified by mortality status MOF: Multi-organ failure; RD: Respiratory dysfunction; ND: Neurologic dysfunction; OP: Other patients. Inter-group differences were assessed using the chi-square test, with results expressed as X2 followed by the degrees of freedom in brackets.

Variables	Survivor (n = 69)	Non-survivor (n = 37)	Statistics	p-value
Baek et al. [9]				
Cluster A	9 (13.0)	13 (35.1)	12.9 [5]	0.024
Cluster B	39 (56.5)	14 (37.8)		
Cluster C	21 (30.5)	10 (27.1)		
Shald et al. [11]				
MOF	28 (40.6)	26 (70.3)	10.4 [3]	0.016
RD	12 (17.4)	6 (16.2)		
ND	9 (13.0)	2 (5.4)		
OP	20 (29.0)	3 (8.1)		
Zhu et al. [12]				
Class 1	25 (36.2)	12 (32.4)	5.43 [6]	0.490
Class 2	5 (7.2)	5 (13.5)		
Class 3	5 (7.2)	5 (13.5)		
Class 4	20 (29.0)	6 (16.2)		
Class 5	6 (8.7)	5 (13.5)		
Class 6	6 (8.7)	4 (18.8)		
Class 7	2 (2.9)	0 (0)		

In the multivariate logistic regression analysis, patients with pneumonia exhibited an 11-fold higher risk of in-hospital mortality than those with other infection sites (adjusted odds ratio [OR], 11.06 [95% CI 3.07-39.82], p < 0.001).

Analyzing the performance of the clinical phenotypic classifications, it was observed that elderly patients presenting with hypothermia showed a significantly higher mortality risk compared to younger, normothermic, or febrile patients (adjusted OR 8.03 [95% CI 1.74-36.98], p = 0.007). Furthermore, patients with multi-organ failure also presented a significantly higher risk of death (adjusted OR 4.16 [95% CI 1.33-12.99], p = 0.014). In contrast, patients who remained persistently hypotensive after 10 hours of hospitalization did not have a statistically significant elevated risk of death (OR, 2.87 [95% CI, 0.546-17.80]; p = 0.257). The results of the univariate and multivariate logistic regression analyses are summarized in Table 4.

**Table 4:** Logistic regression analyses of variables associated with in-hospital mortality OR: Odds ratio; CI: Confidence interval; MOF: Multi-organ failure; RD: Respiratory dysfunction; ND: Neurologic dysfunction; OP: Other patients.

Classification	Univariate analysis OR (95% CI)	p-value	Multivariate analysis OR (95% CI)	p-value
Baek et al. [9]				
Cluster B, C	Reference		Reference	
Cluster A	3.61 (1.36 – 9.55)	0.010	17.32 (1.95 – 153.14)	0.010
Shald et al. [11]				
RD, ND, OP	Reference		Reference	
MOF	3.46 (1.47 – 8.12)	0.040	5.87 (1.17 – 29.948)	0.031
Zhu et al. [12]				
Classes 1, 3, 4, 5, 6, 7	Reference		Reference	
Classes 2	2.00 (0.54 – 7.41)	0.320	3.51 (0.29 – 41.76)	0.300

In the multivariate logistic regression analysis, patients with pneumonia exhibited an 11-fold higher risk of in-hospital mortality than those with other infection sites (adjusted odds ratio [OR], 11.06 [95% CI 3.07-39.82], p < 0.001).

Analyzing the performance of the clinical phenotypic classifications, it was observed that elderly patients presenting with hypothermia showed a significantly higher mortality risk compared to younger, normothermic, or febrile patients (adjusted OR 8.03 [95% CI 1.74-36.98], p = 0.007). Furthermore, patients with multi-organ failure also presented a significantly higher risk of death (adjusted OR 4.16 [95% CI 1.33-12.99], p = 0.014). In contrast, patients who remained persistently hypotensive after 10 hours of hospitalization did not have a statistically significant elevated risk of death (OR, 2.87 [95% CI, 0.546-17.80]; p = 0.257). The results of the univariate and multivariate logistic regression analyses are summarized in Table 4.

**Figure 1:**
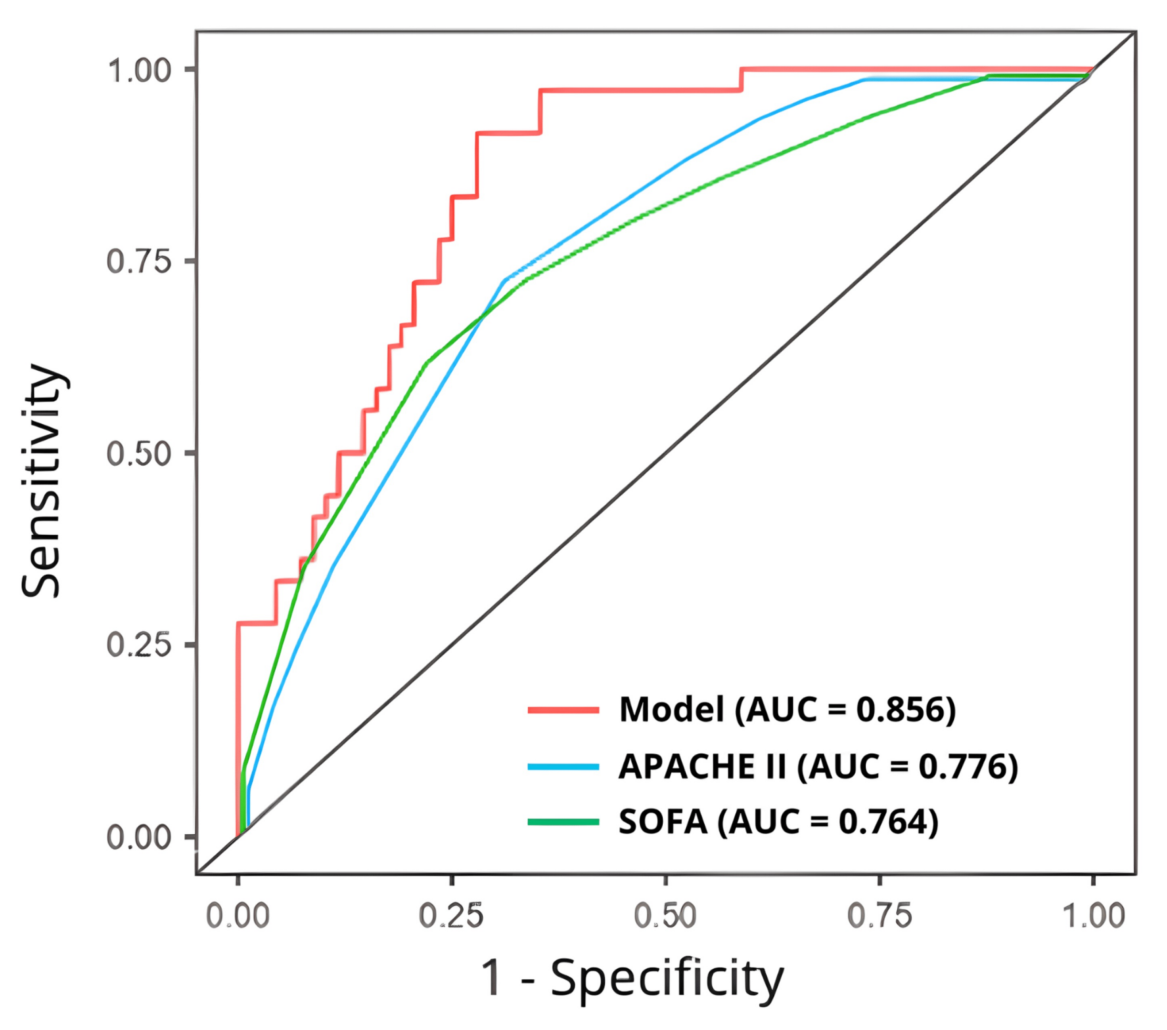
Comparative accuracy of the fitted model with APACHE II and SOFA scores AUC: Area under the receiver operating characteristic curve, APACHE II: Acute physiology and chronic health evaluation II, SOFA: Sequential organ failure assessment

The final predictive model, which integrated all three clinical phenotypic classifications, exhibited non-inferior predictive accuracy for mortality compared to established scoring systems. Specifically, the model achieved an Area Under the Receiver Operating Characteristic curve (AUC) of 0.856, demonstrating superior performance within the studied cohort compared to both the APACHE II (AUC = 0.776) and SOFA (AUC = 0.764) scores. A comparison of the three ROC curves is shown in Figure 1.

Predictive performance of the phenotypic classifications regarding hospital and ICU length of stay

Regarding the Baek et al. classification, no significant differences were observed across groups for either total hospital length of stay (LOS) (χ² = 0.102, df = 2, p = 0.950) or ICU stay (χ² = 0.690, df = 2, p = 0.780).

In contrast, applying the Shald et al. model revealed significant disparities in both hospital and ICU stays (p = 0.05 and p = 0.03, respectively). The median hospital LOS for the RD, MOF, ND, and OP groups was 20, 15, 11, and 10 days, respectively, while the median ICU stay was 11, 9, 7, and 6 days.

Similarly, Zhu’s classification yielded significant differences in hospital LOS (χ² = 16.7, df = 6, p = 0.011) and ICU stay (χ² = 27.3, df = 6, p < 0.001). These findings were particularly pronounced at 10 hours post-ICU admission, with hypotensive patients exhibiting shorter median hospital (11.5 vs. 17.5 days, p = 0.023) and ICU stays (6.0 vs. 11 days, p = 0.011).

## Discussion

This study identified an association among in-hospital mortality due to sepsis, age, and body temperature. Specifically, hypothermia was linked to an increase in the fatality rate, an effect that was significantly more pronounced in elderly individuals.

These findings are consistent with those of previous studies. For example, a meta-analysis evaluating forty-two studies (n = 10,834) reported an inverse correlation between body temperature and mortality in patients [16]. The study observed the lowest fatality rate in febrile patients (22.2%), followed by normothermic (31.2%) and hypothermic patients (47.3%).

The increased mortality risk observed in normothermic and hypothermic patients may be partially attributed to poor adherence to sepsis bundles [17]; however, significant pathophysiological mechanisms likely account for this process. Hyperthermia is an adaptive physiological response that facilitates pathogen clearance; however, hypothermia is typically associated with a maladaptive response, potentially signifying immune exhaustion resulting from prolonged hyperinflammation [8].

Several age-related changes may explain the increased frequency of hypothermia in elderly patients. These include the reduced production of pyrogenic cytokines, such as IL-6 and TNF-alpha, secondary to immune system senescence. Furthermore, decreased muscle mass hinders heat production, and a higher incidence of comorbidities such as stroke can directly impair neurological thermoregulatory mechanisms [18].

This study also identified a significant association between sepsis-related mortality and the presence of MOF. This finding aligns with that of previous research that utilized the same classification system. Specifically, two similar studies by Zhang et al. and Shald et al. observed significantly higher mortality rates in patients with MOF (45.4% and 48.4%, respectively) than in the baseline groups (16.9% and 13.7%) [11,19].

While Zhang et al.'s retrospective analysis of 14,993 septic patients [19] also reported an increased risk of death in the presence of MOF (OR 2.16 [95% CI 1.88-2.47]), the magnitude of this effect was considerably lower than that observed in our study (OR 5.87 [95% CI 1.17-29.94]). This discrepancy in the observed odds ratios is likely attributable to differences in the patient-case mix between the cohorts. Our sample exhibited a higher proportion of males, older age, and greater overall disease severity, as evidenced by a higher mean SOFA score.

Discrepancies exist in sepsis research regarding the methodology used to evaluate organ dysfunction, often owing to the varying emphasis placed on different physiological systems. For example, Knox et al. evaluated pronounced renal impairment in patients with septic shock [10], Ibrahim et al. focused their assessment on cardiogenic and hepatic dysfunction [20], and Ding and Luo highlighted the significance of neurological disease in addition to coagulopathy in their analysis [5].

These variations in the assessment criteria could significantly influence the reproducibility and clinical applicability of the findings across different settings. Consequently, there is currently limited evidence to determine which sepsis phenotype classification based on organ dysfunction offers an optimal prognostic prediction. Ultimately, while most studies emphasize the importance of phenotypic classification in sepsis management, the observed methodological differences highlight a critical need for standardization of the approach to define and quantify organ failure.

Our hemodynamic analysis did not reveal a significant difference in mortality between the groups, exhibiting distinct profiles of SBP variability during the initial 10 hours of ICU admission. These findings contrast those reported by Zhu et al. [12], who observed a significantly worse prognosis in patients with persistently low SBP (40.5% mortality) than in those whose SBP gradually increased from 140 mmHg (11.8% mortality).

The targets employed in the study by Zhu et al. appeared to diverge from the current clinical guidelines for blood pressure management in elderly patients with sepsis. For instance, an extensive British study evaluating 2,600 elderly patients across 65 centers compared a low Mean Arterial Pressure (MAP) target (60-65 mmHg) to standard care. This study concluded that pursuing a lower MAP target was associated with numerically reduced mortality, suggesting the safety of a vasopressor-sparing approach in older adult populations [21].

Finally, our overall statistical predictive model, which incorporated the three phenotypic classifications, demonstrated superior overall predictive ability for in-hospital mortality compared to both SOFA and APACHE II scores. However, the performance of both tools in the present study was lower than the estimates reported in the literature. A comprehensive meta-analysis evaluating 29 distinct studies concluded that the SOFA score remained the optimal predictor of sepsis mortality risk (AUC 0.819 [95% CI 0.783-0.850], p < 0.001), a performance that significantly exceeded that of common serum biomarkers such as procalcitonin and lactate [22]. Similarly, studies consistently reported that APACHE II scores were significantly higher in non-survivors, exhibiting good accuracy in predicting sepsis mortality risk, with reported AUC values generally greater than 0.80 in validation cohorts [23,24].

This discrepancy in performance may be attributed to the differences in our specific patient case mix, potential calibration drift due to evolving clinical practices, or the timing of the initial score calculation. Specifically, the overwhelming demand for ICU beds in the study region often results in prolonged waiting times in the emergency department, thereby introducing a selection bias toward the most critically ill patients. Consequently, the APACHE II and SOFA scores in our cohort were calculated during different time windows compared to most previously published studies. This highly severe patient population could potentially explain the lower discriminatory power of these established tools, as it is inherently more challenging for any scoring system to accurately discriminate between survivors and nonsurvivors within a critically homogeneous group.

This study has a few notable limitations. As with most single-center retrospective cohort studies with a relatively small sample size, our results were inherently limited by compromised external validity because the sample reflects the unique patient demographics (high severity), referral patterns (high demand for ICU beds), and clinical protocols of the specific institution (teaching hospital), thereby restricting the generalizability of the findings to broader or more diverse populations. Internal validity is also threatened by the retrospective design, which is prone to significant information bias stemming from the reliance on existing clinical records.

## Conclusions

In summary, this retrospective cohort study revealed significant disparities in the in-hospital mortality rates across clinical sepsis phenotypes, which were established using a classification scheme based on a combination of age, body temperature, and organ dysfunction severity. Crucially, the study was not adequately powered to detect corresponding differences among the phenotypic classifications based solely on systolic blood pressure (SBP) curves.

Based on these results, clinical sepsis phenotyping is positioning itself as a pivotal area in critical care medicine. To advance this field, future research should focus on refining the characterization of these distinct phenotypic groups using readily available and pragmatic clinical variables.
